# Balanced-detection visible optical coherence tomography with a low-noise supercontinuum laser

**DOI:** 10.1364/BOE.562672

**Published:** 2025-06-23

**Authors:** Lucy Abbott, Gianni Nteroli, Rasmus D. Engelsholm, Patrick Bowen Montague, Adrian Podoleanu, Adrian Bradu

**Affiliations:** 1 Applied Optics Group, School of Engineering, Mathematics and Physics, University of Kent, CT2 7NH, Canterbury, United Kingdom; 2 School of Electrical and Computer Engineering, Technical University of Crete, Greece; 3 NKT Photonics A/S, Blokken 84 DK-3460, Birkerød, Denmark

## Abstract

This paper comprehensively demonstrates the efficiency of balanced detection in a visible optical coherence tomography instrument employing a low-noise supercontinuum laser. By using an innovative technique for digitally aligning camera pixels, we achieved a noise floor reduction of up to 12.8 dB across the entire imaging depth range, particularly near the zero optical path difference between the interferometer arms. The instrument presented here operates at a central wavelength of 590 nm. It delivers high-resolution images with a sensitivity of up to 74 dB in a single spectrometer configuration and 92.8 dB in a balanced configuration. The enhancement in image contrast is exemplified through images of an optical phantom and *in-vivo* images of a human thumb and nail.

## 1. Introduction

Optical Coherence Tomography (OCT) is a non-invasive, three-dimensional imaging modality based on low-coherence interferometry [1]. Applications of OCT are diverse, but in particular, OCT is a recognised standard for retinal imaging and serves as a diagnostic tool for retinal abnormalities and disease progression [2]. OCT has also been utilised to image other biological tissues, such as layers of skin [3], and combined with microscopic capabilities, systems are now being developed for cancer detection [4]. These applications of OCT are conventionally driven by optical sources operating in the near-infrared (NIR). Many optical sources are available at such wavelengths, with most researchers opting for swept sources, especially at wavelengths above 1 µm. Still, only a few research groups have explored using shorter wavelengths in the visible range [5,6,7]. Furthermore, unlike NIR instruments, no commercially available visible (VIS)-OCT instruments are routinely used in clinics.

Although initial reports date back to 2002 [5], the number of applications and publications on VIS-OCT remains quite limited. They typically focus on demonstrating the technology’s technical developments on phantoms [7,8], in animals [9], and in clinical studies that generally involve blood oxygenation measurements in the retina [10,11,12]. The specifications of several recently reported OCT systems listed in Table 1 indicate that visible light illumination at a central wavelength of typically 550-600 nm can achieve a high axial resolution of typically 1-2 µm, although it offers a limited axial imaging depth of only up to 1.8 mm.

**Table 1.** System Specifications of some relevant reported VIS-OCT instruments

Author	Abbott	Wang [15]	Revin [16]	Rubinoff [17]	Pi [11]	Lichtenegger [18]	Yi [13]
Year	2025	2024	2023	2022	2020	2017	2015
Balance	Yes	Yes	No	Yes	No	No	No
Central λ (nm)	590	575	585	560	560	555	564
Range (nm)	75	150	270	94	90	260	115
Axial res. (µm)	3 (air)	1.7 (air)	1.58 (air)	1.7	1.2	0.88	0.97
Depth (mm)	1.6 (air)	1.74	0.85(air)	1.6	1.8	1.8	1.2
Power (µm)	300	220	1000	240	800	800	226
A-scan rate (kHz)	80	120	125	125	50	30	25
Pixel size (µm)	10	10	10	10	-	10	-
Grating (l/mm)	1,800	1800	-	-	-	1800	1800
Sensitivity (dB)	92.8	90.5	75	99.6	90.5	89	86

The VIS-OCT instruments offer two significant advantages over the NIR-OCT ones. First, employing a shorter central wavelength than NIR-OCT facilitates achieving better axial and lateral resolution. As illustrated in the specifications of the instruments shown in Table 1, it can even provide submicron axial resolution, allowing for improved visualisation of various structures within the investigated samples. For example, Yi et al. [13] demonstrated that submicron resolutions enable better separation of layers in the outer segment of photoreceptors and Bruch’s membrane. The second advantage is that VIS-OCT instruments can deliver functional information, which is not obtainable using NIR instrumentation, such as quantifying retinal hypoxia by measuring retinal oxygen delivery and metabolic rates [14].

The limited research on VIS-OCT highlights a significant gap in the existing literature, underscoring the necessity for additional studies and reports on hardware developments of the VIS-OCT instrumentation and its applications

Initially, a prominent shortfall in developing VIS-OCT was attributed to the availability of sources to meet the requirements of a high-quality OCT instrument, such as sufficient optical power in the required spectral range and sufficient bandwidth. The development of supercontinuum (SC) sources delivering enough optical power in the visible range is instrumental in the progression of the field. However, such SC sources have several drawbacks in the practice of OCT. The most critical is excess optical noise dominated by relative intensity noise (RIN) [19], especially in the visible spectral range, which has guided research to apply noise-reduction methods, some previously demonstrated in IR systems [20,21,22], to deliver high-sensitivity images. In particular, this issue has proven the choice of balanced detection OCT (BD-OCT) invaluable to visible systems due to its ability to reduce noise and enhance the signal. As illustrated in Table 1, the balance detection scheme offers better sensitivity than the unbalanced configuration, even when the acquisition rates exceed 100 kHz and with significantly lower optical power on the sample (below 300 µW). For example, Rubinoff *et al*. [17] reported a reduction of 20.5 dB in the noise floor of their BD-VIS-OCT system driven by a SC source. In the near-infrared spectral range, more moderate improvements of 5-10 dB were reported [23,24] for balanced instruments driven by super-luminescent diodes.

The choice of optical components utilised throughout also requires careful consideration due to increased chromatic aberrations at lower wavelengths [7], so most research groups develop in-house rather than utilising commercial spectrometers [6,13], [23–26]. The difficulties in designing identical spectrometers led to the development of digital solutions to compensate for the mismatch between the spectra collected by individual spectrometers [7,27]. In some applications of VIS-OCT, there are strict constraints which must be adhered to. For example, retinal imaging is regulated carefully to prevent optical damage to the eye during the imaging process, and guidelines are set out via ANSI documentation on the safe use of lasers. Typically, up to 250 µW of optical power is used to image the eye with visible light [17] under scanning operation, which is approximately one order of magnitude lower than the permissible optical power when using near-infrared light. This naturally yields low-sensitivity images. To improve the sensitivity of the images, one can increase the optical power incident on the imaged samples, which is possible in some scenarios, such as when imaging skin tissue. For example, it was demonstrated that with 1.0 mW on the sample, sensitivities as high as 91 dB can be produced without needing a balanced detection configuration [16,26]. When the maximum exposure limit is low, or the optical source cannot deliver enough optical power, technical approaches to enhance the sensitivity must be developed.

In early implementations of the balance detection schemes in camera-based OCT instruments, hardware-based approaches were employed to align the two cameras pixel by pixel [21,25]. Due to such adjustment being far from perfect, the remaining differences between the spectrometers limited the reduction in noise floor levels, and most published work frequently reported the benefits of the balance detection scheme in mitigating the autocorrelation terms and fixed pattern noise, rather than improvements in sensitivity. More recent reports [17,26] have introduced techniques for digitally aligning the pixels of the cameras. The advantage of these methods lies in the fact that balanced operation can be efficiently applied to non-identical spectrometers and can thus be employed with wide spectral bandwidth optical sources, which require a large number of pixels in the camera.

Regardless of the technique used to align the pixels of the cameras, generating the BD-OCT images involves a two-step process. The first step consists of calibrating the two cameras (i.e., establishing the distribution of wave-numbers across all pixels and ensuring that the distribution is identical for both cameras). After that, the difference between the spectra collected by the cameras is resampled so that the Fourier Transform (FT) can be applied to data organised along equally spaced wave numbers.

This paper introduces a more straightforward solution for producing BD-OCT images in a single-step process. Based on the Master-Slave (MS) protocol [28,29] to generate OCT images, the subpixel digital alignment of the cameras and the production of the images are achieved by a simple multiplication of matrices. The MS-based technique is implemented in a BD-OCT instrument operating at a central wavelength of 590 nm, and utilising a low optical power on the sample. The instrument features an innovative single diffraction grating dual spectrometer design and incorporates a prototype low-noise supercontinuum laser, the principles of which are explained in Refs. [30–32]: the undertapering principle enables precise control over the second zero-dispersion wavelength, using it as a soliton barrier to effectively halt red-shifting solitons at a predetermined wavelength, thereby locking in the corresponding blue-shifted dispersive waves spectrally and significantly reducing the relative intensity noise (RIN) in the visible spectrum. This supercontinuum laser generates several tens of milliwatts of optical power within our spectral range of interest. However, owing to the in-fibre interferometer configuration and selection of directional couplers, only 300 µW reaches the sample.

## 2. Experimental setup and methodology

### 2.1. Experimental setup

**Fig. 1.**
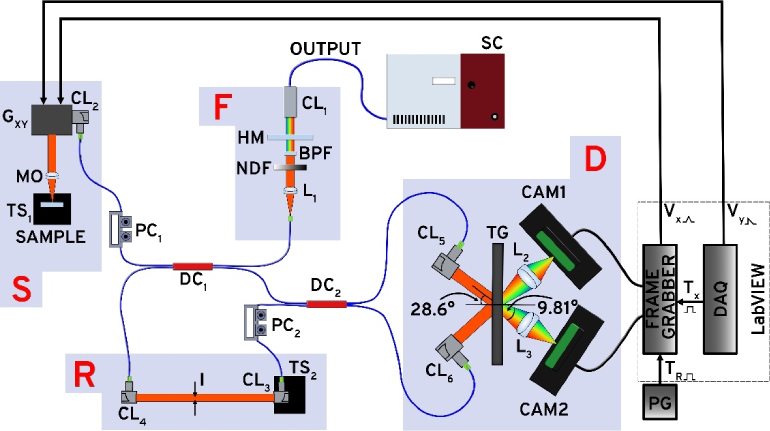
Schematic of the VIS-OCT instrument. F: filter block, S: sample arm block, R: reference arm block, D: detection block. SC: supercontinuum source; PC_1-2_: polarisation controllers; DC_1-2_: directional couplers; CL_1-6_: reflective collimators. HM: hot mirror; BPF: bandpass filter; NDF: neutral density filter; L_1-3_: achromatic lenses; G_xy_: galvanometer scanner; MO: microscope objective; TS_1-2_: translation stage; I: iris; TG: transmission grating; CAM1-2: line scan camera; PG: pulse generator; DAQ: data acquisition card.

Figure 1 depicts a schematic diagram of the balanced OCT instrument. It consists of four main blocks: the filter (F), sample arm (S), reference arm (R), and detection block (D).

#### 2.1.1. Filter block F

A low-noise prototype supercontinuum laser (SC, NKT Photonics, repetition rate 78 MHz) is employed as an optical source. Its output undergoes spectral filtering via block F. A hot mirror with a cut-off wavelength of 1600 nm (Edmund Optics, model 47-304) reduces the amount of IR optical power from the supercontinuum passing through. Following this, a custom bandpass filter (BPF) system featuring a long-pass filter with a 550 nm cut-off wavelength (Thorlabs, model FEL0550) and a short-pass filter with a 625 nm cut-off wavelength (Edmund Optics, model 84-711) is used.

As the SC must operate at full output power to deliver maximum optical power in the visible range, a reflective variable neutral density filter (NDF, Thorlabs, model NDC-50C-4 M) adjusts the optical power across the spectrum. Finally, a 7.5 mm focal length achromatic lens (L_1_, Thorlabs, model AC050-008-A-ML) focuses the filtered light into a fibre patch cord (Thorlabs, 460 HP), which channels the light into the system’s interferometer. The NDF is adjusted so that the optical power at the fibre end, located at L_1_’s focal plane, remains below the fibre’s practical, safe level, as specified by the manufacturer, which is approximately 250 kW/cm^2^, corresponding to about 28 mW in our case.

**Fig. 2.**
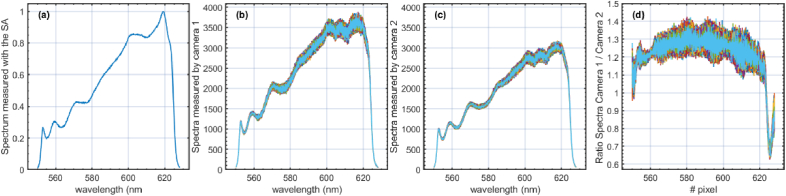
(a) Spectrum measured in the focal plane of MO using an optical spectrum analyser. (b) and (c) 1000 spectra were collected for every 12.5 µs by CAM1 and CAM2, respectively. (d) The ratio between the spectra measured by CAM1 and CAM2.

Figure 2(a) shows an example of a spectrum recorded with an optical spectrum analyser at the focal plane of the microscope objective (MO), where the sample intended for imaging is positioned. This illustrates that our filter block F restricts the supercontinuum spectral range to roughly 80 nm, centred around a wavelength of 590 nm. The spectral range of 550-625 nm ideally aligns with haemoglobin’s strong absorption in the visible range, which is about two orders of magnitude higher than in the NIR. This makes the OCT instrument discussed here an ideal choice for oximetry applications.

#### 2.1.2. Sample arm block S

A custom optical fibre array was assembled, containing a 90:10 ratio directional coupler (DC_1_, Thorlabs, model TW560R2A2) that splits light between the reference and sample arms, respectively, and a 50:50 directional coupler (DC_2_, Thorlabs, model TW560R5A2) that directs the combined beam from both arms to the in-house built spectrometers. In the sample arm, light is directed towards the object to be imaged using a reflective collimator (CL_2_), (Thorlabs, model RC02APC-P01) along with a pair of orthogonal galvanometer mirrors (G_XY_, Thorlabs, model GVS002). A 30 mm achromatic lens (MO), (Thorlabs, model AC254-030-A-ML) focuses the light onto the sample. For calibration and characterisation, a flat silver mirror mounted on a 3D translation stage (TS_1_) optimises the return path of the beam, replacing the object intended for imaging. The optical power in the focal plane of L_1_ was kept at 26 mW, and the light injection efficiency into the small core diameter of 460 HP was 20%. As a result, the maximum optical power measured in the focal plane of MO was 300 µW. The optical power on the sample can be increased by replacing the directional coupler DC_1_ with one of a different splitting ratio or using a bulk beam-splitter.

#### 2.1.3. Reference arm block R

The reference arm uses two reflective collimators (CL_3_ and CL_4_), (Thorlabs, model RC08APC-P01) to launch and collect light. CL_3_ is mounted on a translation stage (TS_2_) for convenient adjustments of the interferometer’s optical path difference (OPD). An adjustable iris diaphragm (I) was employed to adjust the optical power conveyed to the detection block. The lengths of the optical fibres in the array were adjusted to minimise the amount of unbalanced dispersion in the interferometer, aside from that contributed by the thin objective (MO) and the sample itself, thus avoiding additional dispersion compensation blocks.

#### 2.1.4. Balanced detection block D

The detection block D utilises a dual spectrometer which employs a single transmission diffraction grating, in contrast to the conventional two-grating approaches, as seen in previous balanced detection studies [6,21,25,26]. Balanced detection instruments using a single diffraction grating have been reported in [23,24] in a configuration using a single camera where optimal incidence angles for both optical beams incident on the grating cannot be achieved. The use of a single transmission grating is feasible, as incident collimated beams on the grating at ±28.6 degrees are diffracted at approximately ∓45 degrees (with 90 degrees between the first-order diffracted beams), thus providing sufficient space to position large-diameter lenses required by each camera near the grating. This configuration not only offers a cost advantage but, more significantly, simplifies the alignment of the optical components. The diffraction grating in the detection block features a 1800l/mm transmission grating blazed at 532 nm (Wasatch Photonics), with a first-order diffraction efficiency of 50-80% across the spectral range. Two reflective collimators (CL_5_ and CL_6_, Thorlabs, model RC04APC-P01) direct collimated beams onto the grating.

Two lenses (L_2_-L_3_) (Thorlabs, model AC508-100-A), focus the two diffracted beams onto two identical camera-link line-scan cameras, CAM1 and CAM2 (Teledyne e2 V, Octoplus). Out of a single camera’s 2048 pixels, the focused beam covers around 1650 pixels of each camera sensor. As a result, the spectrometers’ resolution is ∼ 0.0485 nm/pixel, yielding a theoretical maximum axial imaging range of 1.7984 mm and a theoretical axial resolution of 3.1 µm in air, a value obtained if it is assumed that the spectra collected by the cameras have a Gaussian shape of width 50 nm. Although the cameras have the potential to operate at 250 kHz, to simplify the instrument by using a single frame grabber (Teledyne Dalsa, model Xtium-CL MX4) and enhance the instrument’s sensitivity, the cameras were externally triggered at a line rate of 80 kHz. Both cameras were operated at minimum amplification gain.

In-house software developed in LabVIEW (National Instruments, Austin, Texas) was used to acquire the data and display real-time images produced by processing data collected by one of the cameras. In post-processing, software was developed in MATLAB (Mathworks, Natick, Massachusetts) and used to digitally remap the spectra collected by one of the cameras and generate BD-OCT images.

**Fig. 3.**
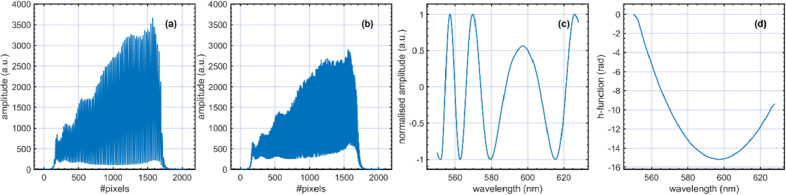
(a) and (b): channelled spectra collected by one of the cameras when as a sample, a flat mirror is used for z = 0.3 mm and z = 0.8 mm, respectively. (c) Normalised channelled spectrum produced when the flat mirror is placed close to z = 0. (d) The h-function (the unbalanced dispersion in the interferometer vs wavelength).

Examples of channelled spectra collected by one of the cameras are presented in Fig. 3(a) and (b). Data to produce these plots was collected with a flat mirror as a sample. The optical powers originating from the sample and reference arms were adjusted to be approximately equal, and the polarisation controllers were adjusted to maximise the visibility of the fringes. In Fig. 3(a), the optical path difference in the interferometer is adjusted to be 0.6 mm (z = 0.3 mm), and in Fig. 3(b), 1.6 mm (z = 0.8 mm). The lengths of the fibres in the interferometer were adjusted to minimise the dispersion left uncompensated by monitoring the number of cycles in the channelled spectrum when z = 0 mm. The minimum number of cycles achieved was four across the spectral range of interest (80 nm covering 1650 pixels), as illustrated in Fig. 3(c), which shows that some residual dispersion is left in the interferometer. This is supported by the calculation of the h-function [28], describing the unbalanced dispersion, which has a parabolic shape (as illustrated in Fig. 3(d)) instead of being constant across the spectral range of interest.

### 2.2. Mapping all pixels and generating A-scans using the MS approach

If Fourier transforms of the channelled spectra are performed to produce A-scans, all pixels of the CAM2 must be mapped. This is because the FT is applied to the difference between the channelled spectra; therefore, both spectra must sample precisely the same k-values. A technique not based on FTs is suggested here, MS [28], which employs raw, not resampled data. The only requirement for the MS technique to produce identical A-scans is that the spectra collected by the cameras span over the same spectral range, with no restriction on how the spectra are sampled within the spectral range. This is due to the property that MS is tolerant to chirp in the spectrum.To generate an A-scan using the MS approach, the product between a theoretically inferred selection of spectra (T) generated before imaging and the channelled spectra as acquired (i.e. not resampled, not corrected, as affected by chirp) is calculated for each camera. The reflectivity at depth “j” is therefore calculated using,

(1) Ajl=|∑
i=1nkl⁡
TijlEil|

**Fig. 4.**
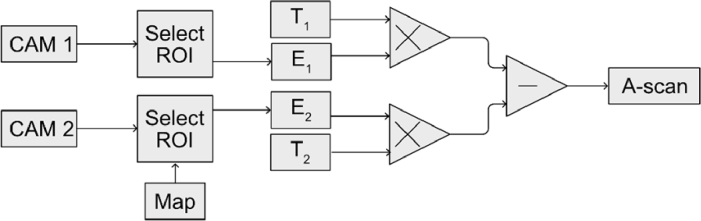
Flowchart of the procedure for producing an A-scan. Channelled spectra collected with cameras CAM1 and CAM2 are first trimmed, keeping only the region of interest (ROI) from each camera (E1 and E2). The spectrum from CAM2 is remapped so that the ROIs span the same spectral range. Then, complex A-scans from each channel are produced by multiplying these spectra, E1 and E2, with the corresponding theoretical complex masks (T1 and T2, respectively). By differentiating the complex A-scans, the final A-scan is obtained.

In Eq. (1), *l* = 1,2 designates one or the other camera. T*^l^*_ij_ are complex 2D arrays of size n*_kl_* × n_z_, where n*_kl_* is the number of sampling points chosen to sample the channels collected by the cameras, and j = 1 .. n_z_, n_z_ is the desired length of the A-scan. E*^l^_i_* are the raw channelled spectra collected by the cameras. The difference between the complex A-scans is calculated to perform the balance operation instead of subtracting the spectra collected by the cameras. A flowchart depicting the procedure of calculating the A-scans in a balanced configuration is presented in Fig. 4.

## 3. Results and discussion

**Fig. 5.**
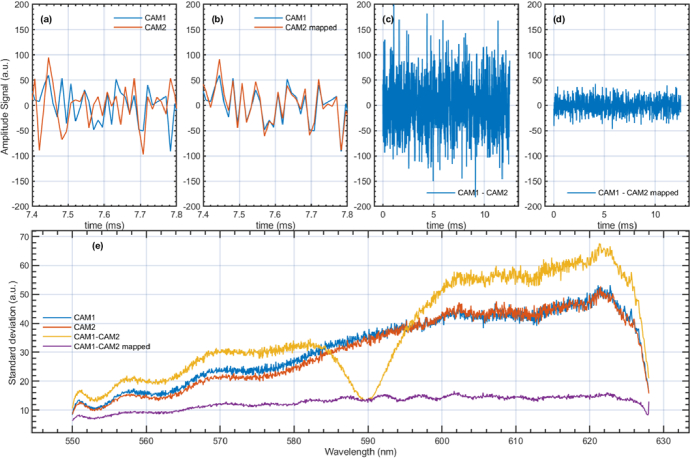
(a) Noise signals collected by the two cameras over 0.5 ms from a pixel. (b) The same as in (a), but the pixels of CAM2 are mapped. (c) Difference between the temporal noise signals collected by the cameras before mapping. (d) Difference between the noise signals after mapping CAM2. (e) Standard deviation calculated for each pixel position for: individual cameras (blue and red), the difference between digitised signals before (yellow) and after (violet) pixel mapping.

The efficiency of using the incoherent noise of the SC to map the pixels of the camera is illustrated in Fig. 5. A zoomed version of the temporal signal collected from pixel **“**i**”** on each camera (corresponding to a wavelength of ∼625** **nm in this case) is presented in Fig. 5(a). As there is no correlation between the two signals, one can conclude that the camera pixels are not correctly aligned, as they are not detecting photons of very similar wavelengths. After mapping the pixels of CAM2, it was found that the temporal signal collected by the pixel **“**i**”** of CAM1 is very similar to that collected by the pixel **“**j**”** of CAM2. This is illustrated in Fig. 5(b), where it can be seen that the temporal noise signals collected by the cameras are nearly identical.

In Fig. 5(c) and 5(b), the difference between the temporal noise signals collected by the cameras before and after mapping the pixels of CAM2 is presented. In this example, the pixel mapping of CAM2 results in a noise reduction of approximately six times (∼13.98 dB) compared to the scenario without mapping.

Figure 5(e) demonstrates to what extent the noise is mitigated in a balanced detection configuration across the entire spectral range using the combined correlation mapping/MS approach. Figure 5(e) shows, in red and blue, the respective values of the standard deviations of the noise signal at each pixel position for CAM1 and CAM2. Kho et al.’s mapping procedure, based on correlating noise signals, was used for five pixels around 550 and 625 nm, which was found to be enough to ensure accurate mapping at the subpixel level. The mapping for the rest of the pixels is automatically achieved using the MS-based technique described in subsection 2.3. The values of the standard deviation of the difference between unmapped spectra (in orange) for the experimental setup are above the values of the individual standard deviation measured by individual cameras. This indicates that the noise signals of different wavelengths are partially in antiphase. The fact that the noise level decreases in the central part of the spectrum without any mapping indicates a good alignment of the cameras across a narrow spectral range in the centre of the spectra. The digital mapping of the spectra collected by CAM2 leads to a drop in the standard deviation of the difference between signals across the entire spectral range (the purple curve) of about 3–5 times.

Pixels around 550 nm and 625 nm were primarily selected due to their positions at the extremities of the optical spectrum being utilised. However, as shown in Fig. 2(a), the optical power level is approximately ¼ of the maximum value at these wavelengths. Even if the optical power is lower at these wavelengths, the excess noise produced by the laser remains incoherent and exhibits unique fluctuation patterns. This is illustrated in the movie presented in Visualization 1 ( Supplement 1). In this movie, the Pearson correlation coefficient is calculated between temporal signals collected from the two cameras. The correlation was calculated between raw signals collected by CAM1 and CAM2 to produce the top plot. In contrast, the correlation between the signals collected by CAM1 and the remapped versions of the signals collected by the second camera is calculated and displayed on the bottom plot. Narrow spectral ranges around 550 and 625 nm are depicted in purple and pink, respectively. As can be seen, around these two wavelengths, the correlation coefficients between the signals collected by CAM1 and the remapped signals collected by CAM2 are above 0.95, indicating the presence of specific fluctuation patterns for each camera at these wavelengths. This is also observable in Fig. S4 ( Supplement 1), where we demonstrate that at 550 and 625 nm, the temporal signals collected by CAM1 and after mapping CAM2 are very similar. Here, we show that the two signals are similar over an extended temporal range (2.5 ms) compared to Fig. 5(b). To illustrate the similarity of the signals over the entire range of 12.5 ms, we have created a second movie in Visualization 2.

The procedure used here to produce A-scans is more straightforward than the one using Fourier transforms. The following steps are typically needed after simultaneously collecting data from the two cameras when using Kho et al.’s mapping procedure together with a Fourier transform-based approach to generate A-scans:

(i) To achieve subpixel mapping, the mapping coefficients for CAM2 are produced by polynomial interpolation of the correlation coefficients across the whole spectral range.
(ii) A cubic B-spline interpolation is typically employed to resample the spectra collected by CAM2 according to the mapping coefficients.
(iii) The difference between spectra collected by CAM1 and the remapped data is produced.
(iv) A second cubic B-spline interpolation is used to resample the difference between spectra according to the non-linearity function g(k) and corrected for unbalanced dispersion according to the pre-processed function h(k).
(v) The absolute value of the FT of the resampled difference of spectra yields a balanced A-scan.

The MS procedure employed here involves:

(i) Mapping pixels only at the edges of the spectra collected.
(ii) Complex A-scans are produced for each camera by multiplying raw, not remapped, or resampled spectra with the corresponding pre-processed theoretical inferred spectra (the procedure of creating A-scans using the MS approach is described in Section 1 of the Supplement 1).
(iii) The absolute value of the difference between the complex A-scans yields a balanced A-scan.

As the FT operates on the difference between the spectra collected by the cameras, the distributions of the wave numbers across the pixels of the two cameras must be identical. This is accomplished by mapping all the pixels of one camera using the computed mapping coefficients. The FT-based approach also requires that the spectra be equally spaced in wave numbers, which is achieved through interpolation using the distribution of the wave numbers, g(k). In contrast, the MS technique does not require any modification of the collected spectra but instead operates on the kernel function of a similar integral calculation to the FT calculation. Therefore, the MS method is immune to the chirp of the spectra since the information about the chirp is embedded in the kernel function. To align the spectra, the MS requires mapping only two pixels of the cameras (from the extremities of the spectra), as long as both spectra span the same spectral range. The number of “digital subpixels” within the spectral range is incorporated into the kernel function. Additionally, the MS approach is very efficient at greater depths and wider bandwidths because the kernel function is inferred using spectra collected at shallow depths, yet it can generate high-resolution and high-sensitivity images across the entire axial range. Our previous work demonstrated that the MS approach can provide better sensitivity and axial resolution at depth than its FT counterpart and is very tolerant of the amount of chirp due to unbalanced dispersion [33,34]. Although we have not experimentally demonstrated this, we do not foresee any limitations in using our approach at greater depths and wider bandwidths.

**Fig. 6.**
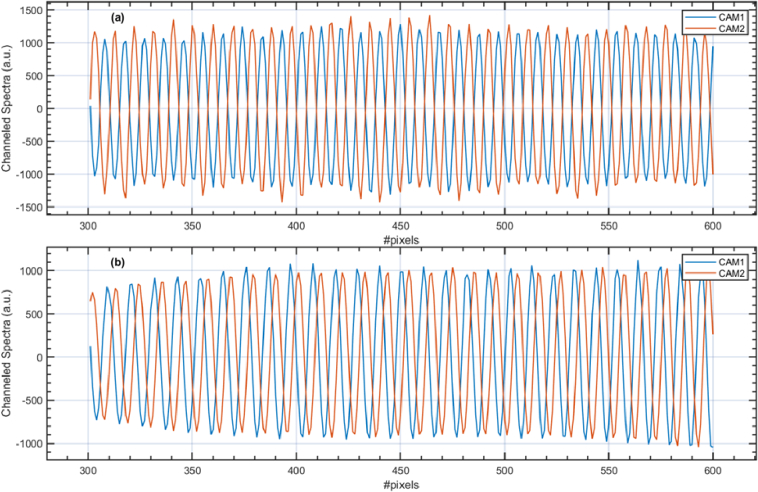
Channelled spectra (a) obtained after automatic remapping of the data; (b) produced when no remapping is performed. The spectra are calculated by inverse Fourier transform of the complex A-scans.

Figure 6 demonstrates that the automatic mapping of the pixels not only leads to a decrease in the noise level, as illustrated in Fig. 6(e), but secures a constant phase difference of π radians across the whole spectral range” between the two interferometric signals, from CAM1 and remapped of CAM2. This property [35] is employed not only by camera-based OCT systems but by swept-source OCT systems as well. The architecture of two splitters in the optics of OCT systems is widely used to enable balanced detection, which is possible due to the phase difference between the outputs of the bulk of fibre splitters. All balance OCT configurations are based on such a property that enables the deduction of deterministic noise and the enhancement of the useful signal. This enables the advantageous operation of balance detection operation, where, by subtracting the two signals, the common path fluctuations and noise are reduced while the signal is increased. The channelled spectra presented in Fig. 6 are generated by calculating the inverse Fourier transform of the A-scans produced using the MS procedure. Only a spectral range covered by 300 camera pixels is shown for better visualisation. The reconstructed channelled spectra presented in Fig. 6 were produced with data collected when a sample flat mirror was placed at an OPD of 0.3 mm

**Fig. 7.**
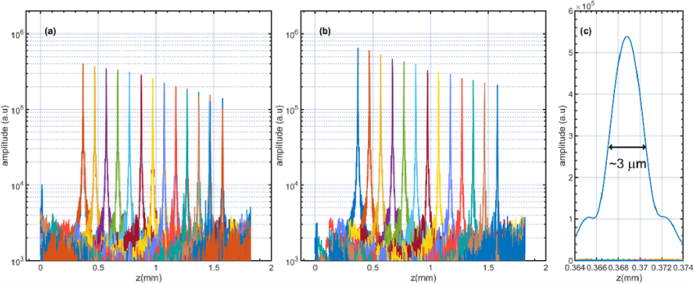
Sensitivity drop-offs for the individual spectrometers (a) and the balance case (b). (c) Zoomed-in version of A-scan presented in (b) showing a maximum at z = 0.35 mm. In (a) and (b), a logarithmic representation is used, whereas in (c), a linear one.

To measure the sensitivity of the instrument, the technique described in [36] was employed. The sensitivity measured when the sample was placed at OPD = 0.7 mm (z = 0.35 mm) was 74 dB for each spectrometer. To produce the sensitivity drop-offs illustrated in Fig. 7, a flat highly reflective metallic mirror placed in the focal plane of the microscope objective MO has been used, for which reason, low-level optical powers illuminated the mirror and convey in the reference arm of the interferometer, to avoid saturating the cameras. A-scans were produced by adjusting the length of the reference arm (by actuating on the position of the translation stage TS_2_). Due to the low power levels, a drop in the noise floor level is not expected by balanced operation. For both situations, unbalanced and balanced, a drop-off of about 6 dB over 1.2 mm can be estimated. The axial resolution was experimentally measured, being defined as the full-width-at-half maximum of the A-scan peaks. As can be seen in Fig. 7(c), where a zoomed version of a single A-scan is shown, the full-width-at-half maximum of the peak is around 3 µm, which indicates that one can achieve axial resolutions in tissue as small as 2.1 µm.

**Fig. 8.**
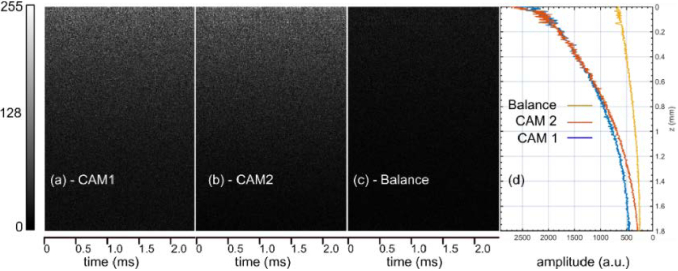
(a), (b) and (c): Noise B-scan images collected with no sample in place were produced with spectra collected from the individual spectrometers and by performing the balance operation. (d) Average of the A-scans presented in (a), (b) and (c). The colorbar shown on the left side of the images corresponds to the levels of grey in (a), (b) and (c).

To analyse the efficiency of the procedure in reducing the noise floor level across the axial imaging range, 200 consecutive spectra were recorded with the sample arm blocked. The iris I (Fig. 1) was used to adjust the optical power incident on the cameras to a grey level close to the saturation of the cameras (∼90% of saturation). Figures 8(a) and (b) show the B-scans generated using the MS approach applied to the spectra individually collected by the two cameras, while in Fig. 8(c), using the procedure depicted in Fig. 5. Figure 8(d) presents the averaged values of the A-scans vs. the axial position z. The fact that the noise floor level is not constant across the imaging range but is decreasing exponentially indicates the presence of excess noise. As it can be noticed, the balance procedure yields lower noise floor levels. However, the excess noise is not entirely rejected. One must note that when the cameras are operated near their saturation limit, as illustrated in Fig. 8, the balance operation reduces the excess noise across the entire imaging range. This is an improvement over other reports [15], demonstrating that the floor approaches the no-excess-noise limit for only around 70% of the imaging range. The noise floor level close to z = 0 is lower when the balance procedure is applied by ∼ 12.8 dB and drops to only ∼6 dB at z = 1.2 mm. Similar drops in the excess noise rejection were observed by Rubinoff et al. [17], who demonstrated that depending on the cameras’ amplification levels, the excess noise rejection could be as large as 20 dB at z = 0 and 10 dB at z = 0.5 mm. Our lower value in noise rejection can be explained by the reduced excess noise exhibited by the SC employed and the slower acquisition rate.

To further prove our instrument’s ability to generate enhanced balanced images, we created B-scan images of a phantom, the thumb, and the nail. In all cases, to increase the sensitivity at depth, we placed the sample so that the focal plane of the MO was inside it. Each image has 500 pixels laterally. Therefore, the data used to create the B-scans was collected in 6.25 ms. The number of axial points across the whole imaging range was equal to twice the number of sampling points of each channelled spectrum; therefore, approximately 0.5 µm per digital point.

**Fig. 9.**
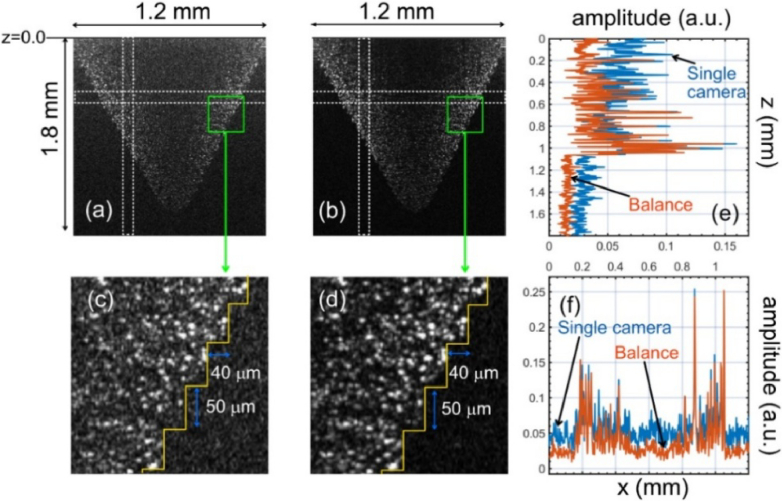
(a) and (b): B-scan images of a pyramid test target obtained by a single spectrometer (a) and after balance operation, respectively. (c) Zoomed in image of the green rectangle from (a). (d) Zoomed in image of the green rectangle from (b). (e) Reflectivity profiles along the z-direction. (f) Reflectivity profiles along the x-direction. The reflectivity profiles in (e) and (f) are from the dashed lines inside the rectangle areas shown in (a) and (b).

**Fig. 10.**
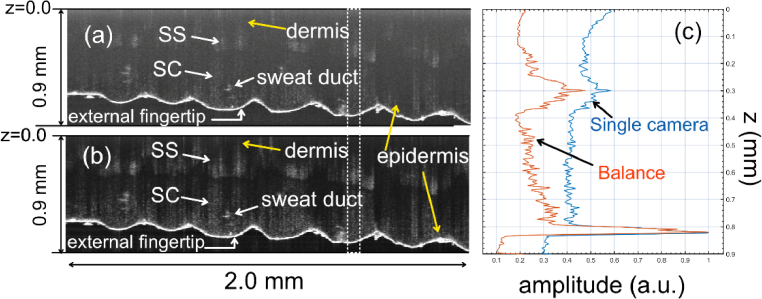
B-scan images of the human thumb, in vivo, obtained using a single spectrometer (a) and after balance operation (b). SC: stratum corneum; SS: stratum spinosum. (c) Reflectivity profiles were collected from approximately where the dashed-bordered rectangle is shown. All distances were measured in the air.

The phantom imaged is a pyramid test target (BioPixS, model OCTPDP01) with a step width of 40 µm and a step height of 50 µm. B-scan images of the phantom obtained with data from a single spectrometer and by balance operations are presented in Fig. 9(a) and 9(b), respectively. Zoomed-in versions of areas of size 320 × 240 µm (green rectangles in Fig. 9(a) and 9(b)) are presented in Fig. 9(c) and 9(d), respectively. As observed, the balance operation improves the image’s contrast (the signal-to-floor noise level ratio). In Fig. 9(d), the edge of the sample is well-defined, and the pyramid’s stairs can be distinguished, whereas in Fig. 9(c), this is not possible due to the larger value of the floor noise level. The fact that the noise floor level drops in the balance situation can be seen and quantified by investigating the reflectivity profiles along the axial (z) or lateral (x)-directions, as illustrated in Figs. 9(e) and 9(f), which were produced simply by using data from 9(a) and 9(b) from the areas shown by the dashed bordered rectangles. When using the balance procedure, it appears clear that at depths above 1.1 mm, the noise floor level drops by about 9 dB than when a single spectrometer is employed (in line with the plots shown in Fig. 8(d)). Near z = 0, the excess noise in a single spectrometer configuration is substantial. As a result, the noise hampers the visibility of the particles in the sample, as illustrated in Fig. 9(c). In Figs. 10(a) and (b), the B-scan images of the human thumb, in-vivo, obtained with the single spectrometer and balance configuration are presented. The axial reflectivity profiles depicted in Fig. 10(c) allow quantifying the level at which the balanced configuration is beneficial. At z = 0.82 mm, at the interface air/skin, the magnitudes of the single spectrometer and balance reflectivities have similar values. However, the contrast when using the balance procedure is better by approximately 6 dB. This improvement is more pronounced at axial positions closer to z = 0. For example, the separation between the stratum corneum (SC) and stratum spinosum (SS) is more evident in the balance configuration than in the single spectrometer configuration. One can estimate, for example, that the contrast of the stratum spinosum (position z = 0. 3 mm) is about 7.36 dB in the balance case and only 1.85 dB in the single spectrometer configuration case.

**Fig. 11.**
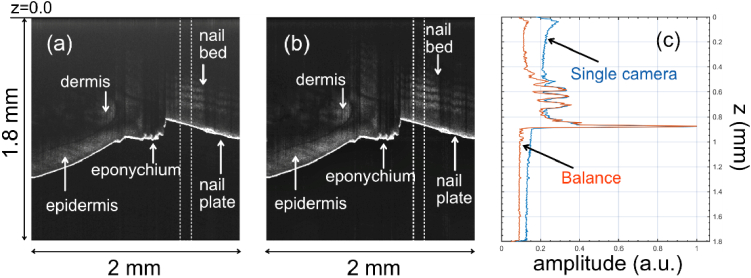
In-vivo B-scan images collected near the eponychium obtained by a single spectrometer in (a) and after the balance procedure (b). (c) Axial reflectivity profiles from the areas shown by the dashed bordered rectangles.

Another illustration of the benefit the balanced approach can bring is illustrated in Fig. 11, where B-scan images around the human eponychium (cuticle) are demonstrated. Again, at around z = 0.82 mm, the magnitude of the reflectivities at the interface air/nail have similar magnitudes; however, the contrast is better when using the balanced approach (∼20 dB vs ∼16 dB). The improvement in contrast closer to z = 0 is noticeable, particularly when examining the “modulations” caused by the layers inside the nail, at approximately z ∼ 0.6 mm, where we can estimate that the contrast of the layers is about 7.4 dB for the balance configuration and 4.0 dB for the single spectrometer configuration.

## 4. Conclusion

This paper demonstrates the superior effectiveness of balanced detection in a visible Optical Coherence Tomography instrument using a low-noise supercontinuum laser. By implementing an innovative method for digitally aligning the cameras’ pixels, the noise floor level was substantially reduced by up to 12.8 dB, throughout the entire imaging range compared to non-balanced detection devices. The instrument operates at a central wavelength of 590 nm, delivering high-resolution images with a sensitivity of 74 dB in a single spectrometer configuration and 92.8 dB in a balanced configuration. Images of an optical phantom and *in-vivo* images of a human thumb and nail illustrated the enhancement in image contrast.

The successful implementation of the MS-based technique for digitally aligning the pixels of the cameras requires only that the spectral range sampled by the cameras be the same, without prior knowledge of the wave numbers associated with the first and last sampled pixels. In addition, there is no need of any knowledge about the distribution of the wave numbers between the first and last pixels of the camera. The MS technique operates on raw, unresampled data by altering the kernel function instead of the collected spectra, as detailed in Section 1 of the Supplement 1. The mapping of the first and last pixels was achieved by using the method proposed by Kho *et al*. [26] (detailed in the Supplement 1 as well), but can also be accomplished using alternative methods to calculate the distribution of wavelengths across the camera’s pixels, such as direct calculation from the geometry of the spectrometers or the use of excitation with narrow spectral lines. We opted for Kho’s method to map the pixels at the extremities of the spectra due to its robustness, which does not require narrow spectral lines at exotic wavelengths.

To ensure automatic digital alignment of the camera pixels with sub-pixel resolution, the MS technique was utilised, enabling the generation of A-scans without prior knowledge of the wave-number distribution across the camera pixels. This streamlined the data processing process of generating balanced images. The process requires only the subtraction of complex axial reflectivity profiles obtained from data collected by the two spectrometers, thus eliminating the need for multiple interpolations. This method reduced the noise floor level across the full imaging range and so outperformed existing reports, which typically show improvements in only up to 70% of the imaging range. At shallow depths, our MS-based technique for digitally aligning the spectra reduces the noise floor level by the same amount as the method used by Kho et al. when both approaches are applied to data collected by our instrument. A comparison with data presented in Kho’s paper or reports from other research groups using Kho’s method cannot be made due to the many variables that differentiate our setups (different sources, acquisition rates, cameras, etc.). Moreover, as illustrated in the benchmarking presented in the Supplement 1, the technique we proposed here is computationally less expensive than other techniques involving the use of Fourier transforms and has the potential to generate balanced images in real-time, as the only operation required after digitisation of the spectra is a simple matrix multiplication, rather than two sequential interpolations of the digitised spectra.

However, the reduction in noise floor level achieved with our instrument is lower than that reported in other studies, owing to the lower-noise supercontinuum source and the longer acquisition rates we employed.

Regarding the robustness of the MS alignment procedure, we have not noticed any inconsistencies in our data requiring instrument recalibration. However, we recognise that, since the approach proposed here relies on mapping pixels, any camera drift due to any cause, including environmental changes, will necessitate redoing the mapping and recomputing the kernel function. Nevertheless, the same drift equally affects any other mapping procedure.

## Supplemental information

Supplement 1 Supplement 1 https://doi.org/10.6084/m9.figshare.29313014

Visualization 1 Correlation coefficients at the extremities of the spectra https://doi.org/10.6084/m9.figshare.28920836

Visualization 2 Noise signals before and after mapping over 12.5 ms https://doi.org/10.6084/m9.figshare.28920851

## Data Availability

Data underlying the results presented in this paper are not publicly available at this time but may be obtained from the authors upon reasonable request.

## Supplemental document

See Supplement 1 for supporting content.
