# Supplementary material for: Balanced-detection visible optical coherence tomography with a low-noise supercontinuum laser
Source: Biomed Opt Express. 2025 Jun 23;16(7):2898–913. doi: 10.1364/BOE.562672 (PMC12265444; doi:10.1364/BOE.562672)
Supplement: Supplementary file 2 [file boe-16-7-2898-s001.pdf]

Supplemental Document

Biomedical Optics EXPRESS

Balanced-detection visible optical coherence tomography with a low-noise supercontinuum laser: supplement

LUCY ABBOTT,[1] GIANNI NTEROLI,[1,2] RASMUS D. ENGELSHOLM,[3] PATRICK BOWEN MONTAGUE,[3] ADRIAN PODOLEANU,[1] AND ADRIAN BRADU[1,*]

[1]*Applied Optics Group, School of Engineering, Mathematics and Physics, University of Kent, CT2 7NH, Canterbury, United Kingdom*
[2]*School of Electrical and Computer Engineering, Technical University of Crete, Greece*
[3]*NKT Photonics A/S, Blokken 84 DK-3460, Birkerød, Denmark*
[*]*a.bradu@kent.ac.uk*

This supplement published with Optica Publishing Group on 23 June 2025 by The Authors under the terms of the Creative Commons Attribution 4.0 License in the format provided by the authors and unedited. Further distribution of this work must maintain attribution to the author(s) and the published article's title, journal citation, and DOI.

Supplement DOI: https://doi.org/10.6084/m9.figshare.29313014

Parent Article DOI: https://doi.org/10.1364/BOE.562672

Balanced-detection visible optical coherence tomography with a low-noise supercontinuum laser

LUCY ABBOTT,[1,] GIANNI NTEROLI,[1,2] RASMUS D. ENGELSHOLM,[3,] PATRICK BOWEN MONTAGUE,[2,] ADRIAN PODOLEANU,[1,] AND ADRIAN BRADU[1]

Supplementary Materials

1. Fourier Transform (FT) versus Master Slave (MS) based reflectivity profiles

1.1 Fourier Transform reflectivity profile

A Fourier transform reflectivity profile, *A(z)*, is produced by calculating the Fourier transform of the digital spectra (*E(k)*) at the output of the frame grabbers or digitisers employed, which is the integral of the product between *E(k)* and the kernel function e^{jkz}:

$$A(z) = \mathrm{FT}[w(k)E(k)] = \int_{-\infty}^{+\infty} w(k)E(k)e^{jkz}dk \tag{S1}$$

Here, w(k) is an apodisation function. The above equation can lead to a high-resolution A-scan if the sampling coordinate k (the wavenumber) is linear. In most of the implementations, however, the wavenumber is described by a nonlinear function $g(k) \neq k$, and as a result, the spectra E must be resampled according to a linear distribution, $\hat{k}$, by employing a cubic B-spline interpolation. In addition, to compensate for the unbalanced dispersion, h(k), between the arms of the interferometer, the resampled function E must be multiplied by $e^{-jh(\hat{k})}$. As a result, an A-scan is produced using,

$$A(z) = \mathrm{FT}\left[w\left(\hat{k}\right)E\left(\hat{k}\right)\right] = \int_{-\infty}^{+\infty} w\left(\hat{k}\right)E\left(\hat{k}\right)e^{j\hat{k}z}e^{-jh\left(\hat{k}\right)}d\hat{k} \tag{S2}$$

The calculation of the two functions, g(k) and h(k), is well documented in various papers [S1-S4].

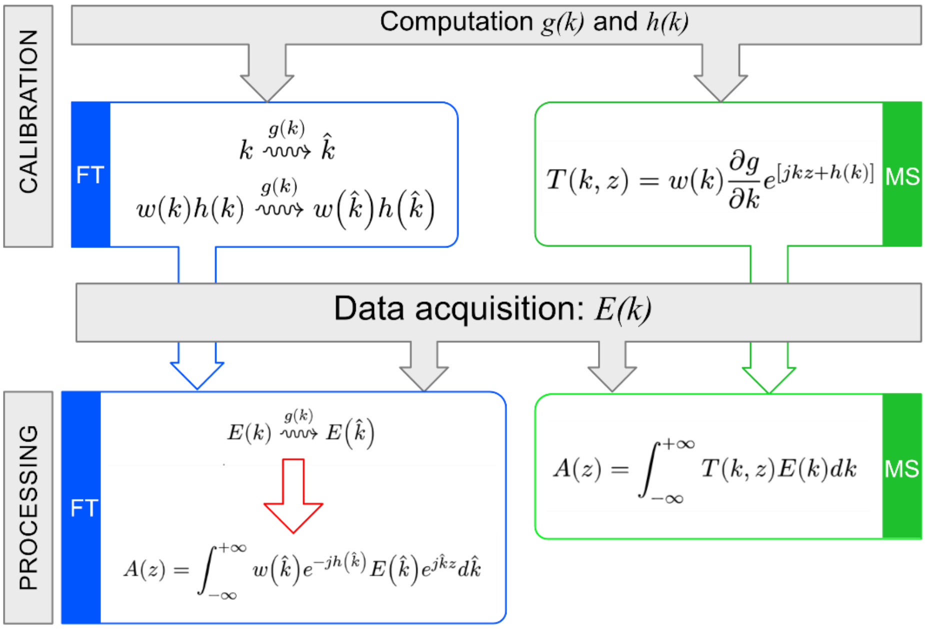

Fig. S1 A flowchart illustrating generating A-scans using the FT (left, in blue) and MS (right, in green). At the calibration stage, the functions g(k) and h(k) are used to resample the h-function (when FT is used) and infer the complex theoretical spectra (when the MS is used). After data acquisition, the FT requires resampling the spectra and the calculation of their Fourier transform. The MS requires only calculating the product between the inferred theoretical and experimental spectra.

1.2 Master-slave based reflectivity profile

The MS technique also relies on calculating an integral, but unlike the FT method, it modifies the kernel function instead of resampling the spectra. For each axial position z, the integral of the product between a complex, theoretically inferred spectrum, T(z,k), and the experimental digital spectrum is calculated:

$$A(z) = \int_{-\infty}^{+\infty} T(k,z)E(k)dk \tag{S3}$$

The theoretically inferred spectrum can be computed using [S2-3],

$$T(k,z) = w(k)\frac{\partial g}{\partial k}e^{[jkz+h(k)]} \tag{S4}$$

i.e. chirped exponentials replace the harmonic functions in S2. The advantages of the MS approach are that it does not involve computationally expensive procedures due to the resampling of the spectra and allows for the production of A-scans covering desired axial regions of interest. The number of sampling points within the image axial range of interest (the number of z values) is embedded into the kernel function. This is in contrast to the FT method, which always provides a fixed axial range, and the number of axial points can be adjusted via zero-padding. The MS approach is very efficient at higher depths/wider bandwidths because the kernel function is inferred using spectra collected at shallow depths, but usable to generate high-resolution and high-sensitivity images across the entire axial range. Our previous work demonstrated that the MS approach can provide similar or even better sensitivity and axial resolution at depth than its FT counterpart [S3], and it is tolerant to the amount of chirp in the collected spectra due to the unbalanced dispersion in the interferometer [S4].

In Fig. S1, a flowchart diagram shows the steps required to produce A-scans using the two techniques.

The mathematical calculations needed for generating B-scans involve resampling each of the acquired spectra, followed by their Fourier transform (FT method), or the multiplication of the acquired and theoretically inferred spectra (MS method) [S2-S4]. Depending on the acquisition rate and the number of sampling points of the digitised acquired spectra, both approaches have the potential to provide images in real-time. However, the MS method does not involve data resampling; therefore, it is, in general, computationally less expensive than the FT method. To illustrate this, a LabVIEW 2018 (National Instruments, Austin, Texas) project was created and run on the computer used to acquire the data presented in the main document. The computer was equipped with an Intel Xeon W-2265 CPU at 3.5 GHz and 32 GB of RAM.

Our findings are summarised in Fig. S2, which presents the time required to produce B-scans composed of 500 A-scans across various scenarios. Given that the acquisition rate of the cameras used in our study was 80 kHz, the 500 spectra necessary for producing one B-scan were collected within 6.25 ms. The real-time operation (illustrated by the green box in Fig. S2) is ensured if the data processing time is below 6.25 ms.

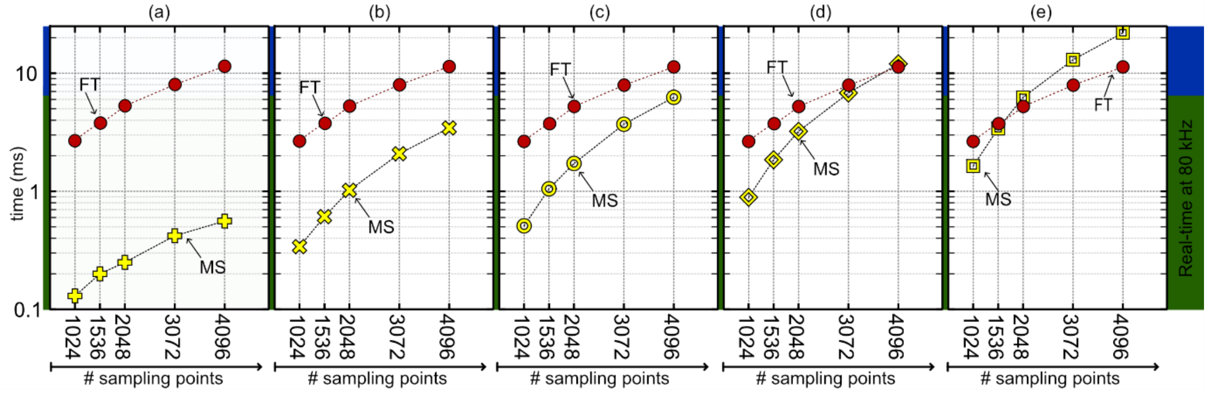

Fig. S2: Time to produce B-scan images using the FT (red filled circles) and MS (yellow filled shapes) based methods as a function of the number of sampling points N_k. The number of points along the depth when employing the FT method is always $N_k/2$. When using the MS method, the B-scan images have one axial point for (a), $N_k/16$ in (b), $N_k/8$ in (c), $N_k/4$ in (d), and $N_k/2$ for (e).

When using the FT technique to produce images, the number of axial points is half the number of sampling points, N_k, into which the spectra are sampled (unless an additional interpolation technique is employed). In contrast, the MS method allows for the generation of a variable number of axial points, at the user's discretion

[S4]. The number of sampling points (the number of camera pixels) in our setup was N_k = 2048, for which the processing time when using FT is 5.26 ms, and 6.2 ms when using MS (Fig. S2(e)). Therefore, both methods can generate one B-scan in real-time.

In a balanced configuration, however, due to the larger number of spectra that need to be processed, neither method operates in real-time unless (i) a CPU with superior computational capabilities is utilised or (ii) the number of axial points is reduced. By reducing the number of axial points to $N_k/4$, the processing time for a single B-scan is lowered to approximately 3 ms when using the MS method, thereby achieving real-time operation. Although our cameras were operated at 80 kHz, they could potentially be run at 250 kHz; in which case, real-time operation is only feasible if the processing time is below 2 ms. When using the FT-based method, even with the number of sampling points reduced to 1024, real-time operation cannot be achieved. However, the MS method delivers real-time B-scans with $N_k/8$ axial points.

2. The procedure of pixel mapping

While there was a significant attempt to align the two spectrometers in hardware, achieving perfect alignment across the entire spectral range proved challenging. To effectively align the cameras, we implemented a digital alignment approach similar to the technique suggested by Kho et al. [S5], which uses the fact that the excess noise produced by any supercontinuum laser is incoherent, i.e., it has a unique fluctuation pattern for each wavelength. As a result, each camera pixel has its particular temporal print. However, instead of mapping all the camera's pixels to the corresponding pixels of the other camera, we only mapped the pixels at the extremities of the spectra. As discussed below, there is no need to map the other pixels or know the noise fluctuation patterns for all pixels when the MS technique of generating an A-scan is employed.

In general, to implement the procedure of mapping the pixels, N-noise spectra are collected simultaneously by the two cameras, $C_1(k, t)$ and $C_2(k, t)$. Here, k refers to the wavenumber corresponding to a particular camera pixel due to dispersion in the spectrometer, whereas t is time. To map the pixels, the Pearson correlation coefficient between temporal noise signals has been calculated using the formula below,

$$M_{i,j} = \frac{1}{N-1}\sum_{l=1}^{N} \frac{C_1(k_i,t_l)-\overline{C_1}}{\mathrm{std}(C_1)} \cdot \frac{C_2(k_j,t_l)-\overline{C_2}}{\mathrm{std}(C_2)} \qquad \text{(S5)}$$

When mapping all N_k pixels of the camera, i = 1,2, …., N_k and j = 1, 2 … N_k; however, here we only calculated the correlation coefficient for 5 pixels around each of two wavelength values of 555 and 625 nm. The procedure of mapping the pixels of the two cameras is presented in the flowchart shown in Fig. S3. The two cameras, operating at 80 kHz, synchronously collected N=1,000 consecutive spectra similar in shape to those presented in Fig. 2(b) in the main manuscript and Fig. 2(c). As the amplitudes and the shapes of the spectra collected by the two cameras were not similar due to the imperfect alignment and the deviation from 50/50 of the splitting ratio of DC_2, a correction factor defined as the ratio between the averaged spectra collected by the cameras (similar to that presented in Fig. 2(d) in the main manuscript) was applied to the spectra collected by CAM2. Then, the temporally averaged signal value was deducted for each k-pixel to improve the efficiency of the procedure. Finally, the Pearson correlation coefficients were calculated, and the pair of pixel indices (i, j) for which maxima were found was retained.

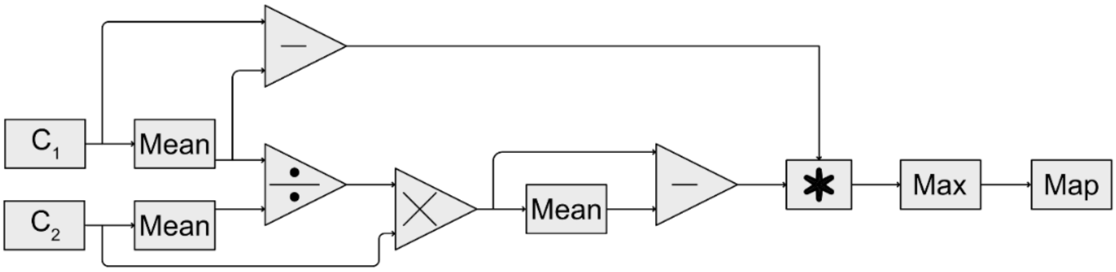

Fig. S3: Flowchart showing the procedure employed to create the correlation map. C_1 and C_2 are selections of spectra collected by the two cameras in synchronism. The "Mean" block performs an average of the spectra. The ÷, ×, and – blocks produce the ratio, multiplication, and difference between spectra along the coordinate k, for each pixel pair. The * block calculates the correlation between spectra. The "Max" block searches for the maximum value of the correlation vector.

3. Mapping pixels at the extremities of the spectrum

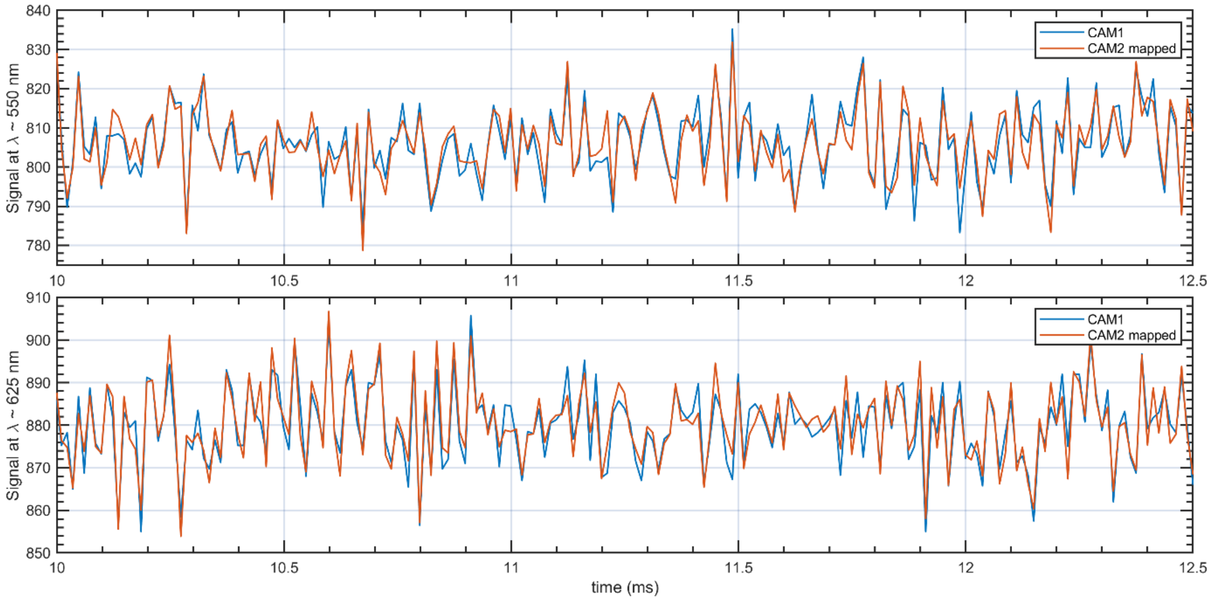

Fig. S4: Noise signals collected by CAM1 (red) and CAM2 after pixel remapping (blue) over a 2.5 ms. The top figure illustrates the noise signals from the two camera pixels at ~ 550 nm, whereas the one at the bottom from ~ 625 nm. To demonstrate the efficiency of the mapping procedure across the entire 12.5 ms over which the 1,000 spectra were collected, a video (Visualisation 2) has been created.

Supplementary references

S1. S. Makita, T. Fabritius, and Y. Yasuno, "Full-range, high-speed, high-resolution 1 μm spectral-domain optical coherence tomography using BM-scan for volumetric imaging of the human posterior eye," Opt. Express **16**, 8406–8420 (2008).

S2. S. Rivet, A. Bradu, F. Bairstow, *et al*., "Group refractive index and group velocity dispersion measurement by complex master slave interferometry," Opt. Express **26**, 21831-21842 (2018).

S3. A. Bradu, N.M. Israelsen, M. Maria, *et al*., "Recovering distance information in spectral domain interferometry," Scientific Reports **8**, 15445 (2018).

S4. A. Bradu, M. Maria, and A. Podoleanu, "Demonstration of tolerance to dispersion of master/slave interferometry," Opt. Express **23**, 14148-14161 (2015).

S5. Kho, A.M., Zhang, T., Zhu, J. *et al*., "Incoherent excess noise spectrally encodes broadband light sources," Light Sci. Appl. **9**, 172 (2020).
